# Comparison of isolated tenotomy vs. tenotomy with tenodesis for long head of biceps tendon in middle-aged and elderly patients undergoing rotator cuff repair: a retrospective study

**DOI:** 10.3389/fsurg.2025.1727578

**Published:** 2026-01-12

**Authors:** Ning Ma, Xinghua Chen, Chenshuai Pan, Zonggang Xie

**Affiliations:** 1 Department of Orthopedics, The Second Affiliated Hospital of Soochow University, Suzhou, China; 2 Department of Orthopedics, Taizhou Central Hospital (Taizhou University Hospital), Taizhou, Zhejiang, China; 3 Department of Orthopedics, The People's Hospital of Yuhuan, Taizhou, Zhejiang, China

**Keywords:** isolated biceps tenotomy, long head of the biceps tendon, rotator cuff tears, tenotomy with tenodesis, treatment

## Abstract

**Purpose:**

Rotator cuff tears (RCTs) are a prevalent source of shoulder disability, frequently accompanied by pathologies of the long head of the biceps tendon (LHBT). While both tenotomy and tenodesis are established procedures for managing LHBT lesions during rotator cuff repair, their comparative efficacy remains a subject of debate. This study aimed to compare the clinical outcomes of isolated tenotomy vs. tenotomy with tenodesis in patients undergoing arthroscopic RCTs in a retrospective study.

**Methods:**

All surgical procedures involved arthroscopic rotator cuff repair performed by a single surgeon. Patients were divided into isolated biceps tenotomy and tenotomy with tenodesis using a suture anchor. Postoperative rehabilitation was standardized. Outcomes were assessed preoperatively and at 3, 6, 12 months, and final follow-up using ASES and Constant-Murley scores, VAS pain scale, operative time, and complications.

**Results:**

A total of 63 patients (mean age 67.3 years) were retrospectively reviewed and divided into two groups: isolated tenotomy (*n* = 28) and tenotomy with tenodesis (*n* = 35). Preoperative demographics and functional scores (ASES, Constant-Murley, VAS) were comparable between groups. Both techniques resulted in significant and sustained improvements in all functional and pain outcomes at 3, 6, and 12 months postoperatively compared to baseline. In the early postoperative period (3 months), the tenotomy group demonstrated a statistically superior improvement in VAS pain scores. However, these differences in functional and pain scores between the two groups were no longer significant at the 6 and 12-month follow-ups. The operative time was significantly shorter for the tenotomy group. The only significant complication difference was the occurrence of Popeye deformity in two patients (14%) in the tenotomy group, with no cases in the tenodesis group.

**Conclusion:**

In conclusion, both isolated tenotomy and tenodesis provide equivalent, excellent functional improvements and pain relief at the 12-month follow-up. Tenotomy offers the advantages of a shorter operative time and better early pain control, at the cost of a higher risk of Popeye deformity. Tenodesis effectively prevents this cosmetic complication but requires a longer surgery.

## 1 Introduction

Rotator cuff tears (RCTs), a prevalent cause of shoulder pain and functional impairment, represent a prevalence of 20% of the general population, especially in middle-aged and elderly populations, significantly diminishing quality of life (1). A substantial proportion of patients with RCTs exhibit concomitant pathologies of the long head of the biceps tendon (LHBT), which is increasingly recognized as a contributor to persistent shoulder pain and dysfunction even after successful rotator cuff repair (2). The close anatomical and functional relationship between the LHBT and the rotator cuff makes it a critical structure to address during surgical intervention (3). Traditional management strategies for LHBT lesions include tenotomy (simple tendon release) and tenodesis (tendon release followed by reattachment to the humerus) (4). Tenotomy is technically simpler, requires less operating time, and facilitates quicker rehabilitation (5). However, it carries a risk of Popeye deformity, muscle cramping, and potential slight loss of elbow flexion strength. In contrast, tenodesis aims to maintain the length-tension relationship of the biceps muscle, potentially reducing the risk of cosmetic deformity and preserving strength, but it involves a more complex procedure, potentially longer surgery time, and may be associated with implant-related complications such as anchor loosening or infection (6).

With advancements in arthroscopic techniques, the debate regarding the optimal treatment for LHBT lesions during rotator cuff repair remains unresolved, particularly in the middle-aged and elderly demographic. Recent studies, including randomized controlled trials, have sought to compare these techniques. The LOOPTEN trial (2025), a multicenter non-inferiority RCT, demonstrated that implant-free loop tenodesis was non-inferior to arthroscopic anchor tenodesis in functional and cosmetic outcomes while avoiding implant-related risks (7). However, other studies have reported conflicting findings. Some investigators suggested superior early pain relief with tenotomy, while others indicated better functional outcomes and lower complication rates with tenodesis in the long term (8).

The purpose of this retrospective study was to compare the clinical outcomes, including functional scores, pain relief, operation time, and complication rates (particularly Popeye deformity), between isolated tenotomy and tenotomy with tenodesis for LHBT lesions in middle-aged and elderly patients undergoing arthroscopic rotator cuff repair. This research aims to provide further evidence to guide surgical decision-making in this common clinical scenario.

## 2 Methods

### 2.1 Study design and patient selection

A retrospective analysis was conducted on 63 elderly patients with rotator cuff tears and concomitant LHBT pathologies who underwent arthroscopic surgery at our institution between January 2020 and December 2022. The patients were divided into two groups based on the surgical procedure performed on the LHBT: an isolated tenotomy group (28 patients) and a tenotomy with tenodesis group (35 patients).

Inclusion criteria were: (1) in this study, we aimed to investigate the sample on middle-aged and elderly, so we only include those age ≥50 years; (2) symptomatic full-thickness rotator cuff tear confirmed by MRI; (3) associated LHBT lesion (e.g., tendinitis, partial tear, subluxation, or degeneration) diagnosed preoperatively by physical examination (e.g., Speed test, Yergason test) and confirmed arthroscopically; (4) completion of a minimum 12-month follow-up. In this study, the exclusion criteria included: (1) previous shoulder surgery; (2) massive, irreparable rotator cuff tears (assessed intraoperatively based on tissue quality and mobility); (3) glenohumeral osteoarthritis (Samilson & Prieto grade II or higher); (4) neuromuscular disorders affecting the shoulder; (5) infection; or (6) inadequate medical records. This study was approved by the Institutional Review Board of Taizhou Central Hospital (Taizhou University Hospital, Reference No. 2021K-07-04). All patients provided written informed consent before surgery.

### 2.2 Surgical technique

All procedures were performed by the same senior orthopedic surgeon with extensive experience in shoulder arthroscopy. Patients received general anesthesia and were placed in the beach-chair position. Standard posterior and anterior arthroscopic portals were established.

Diagnostic arthroscopy was performed first to thoroughly evaluate the glenohumeral joint, including the rotator cuff tendons, labrum, and especially the LHBT (assessing for inflammation, tearing, instability, or degeneration). Subsequently, rotator cuff repair was performed using suture anchors after necessary debridement and mobilization.

#### 2.2.1 For the LHBT

**Figure 1:**
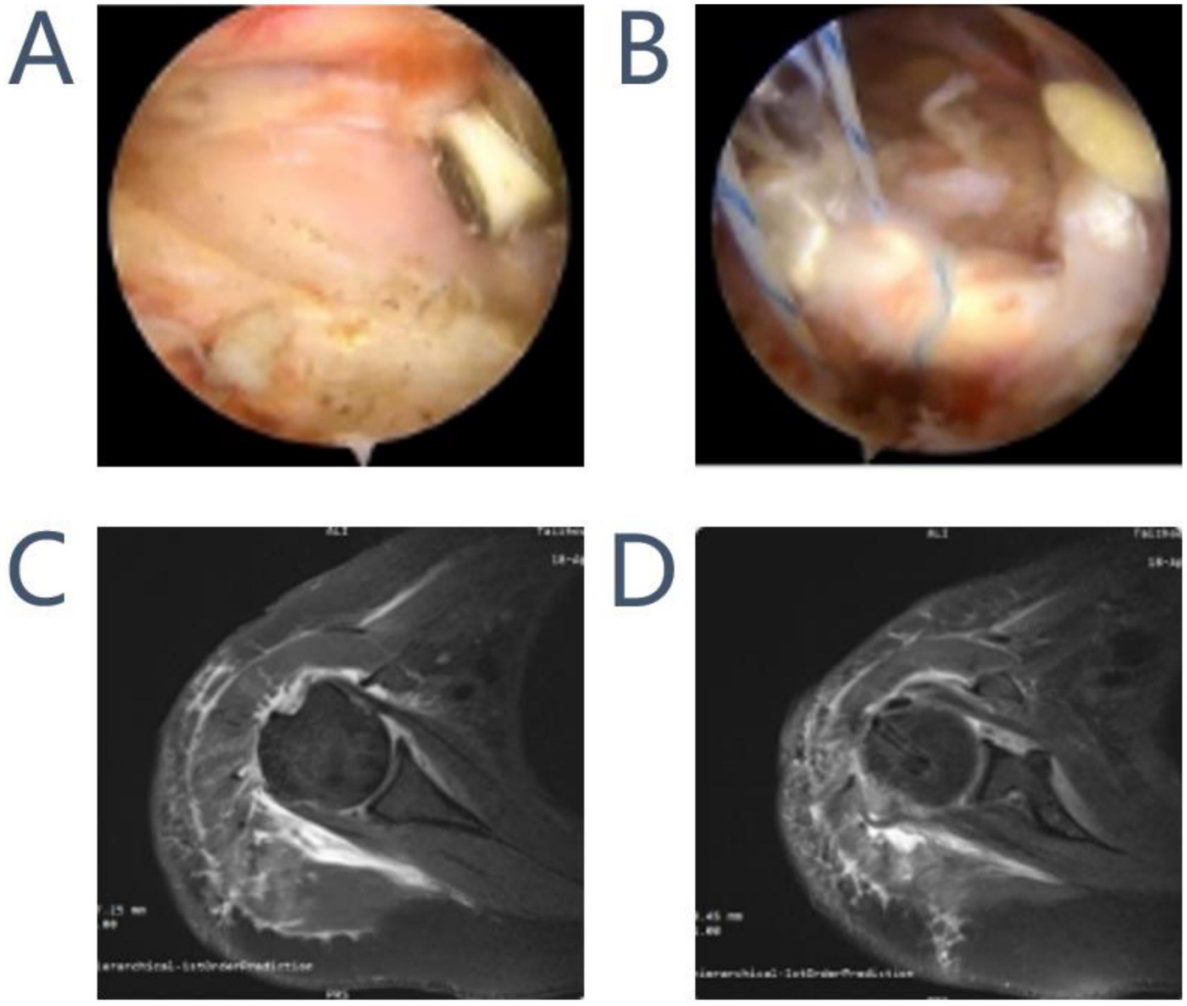
Intraoperative transection of the long head of the biceps brachii tendon **(A)** and fixation of the long head of the biceps brachii tendon (at the intertubercular groove level) **(B)** postoperative MRI images of the shoulder joint following transection of the long head of the biceps brachii tendon **(C)** and the shoulder joint following fixation of the long head of the biceps brachii tendon **(D).**

Isolated Tenotomy Group: The LHBT was released at its origin from the supraglenoid tubercule and labrum using an arthroscopic cutter. The tendon was allowed to retract distally into the bicipital groove.
Tenotomy with Tenodesis Group: After arthroscopic tenotomy at the origin, the intra-articular portion of the LHBT was resected. The tendon was mobilized and fixed in the bicipital groove at the level of the superior border of the pectoralis major muscle using a suture anchor technique. The tendon stump was sutured and secured to the anchor, ensuring adequate tension and stability. Figure 1 showed the surgery and the post-operation with the MRI features of Isolated Tenotomy Group and Tenotomy with Tenodesis Group.

After surgery, all patients were immobilized in an abduction brace for 6 weeks. Passive range-of-motion exercises began on the second postoperative day. Active assisted and active exercises were initiated at 6 weeks postoperatively, followed by progressive strengthening exercises after 12 weeks.

### 2.3 Outcome measures

Patients were evaluated preoperatively and postoperatively at 3, 6, 12 months, and at the latest follow-up. The primary outcome measures included the follow items. Functional Scores: American Shoulder and Elbow Surgeons (ASES) score and Constant-Murley shoulder score. Pain Assessment: Visual Analog Scale (VAS) for pain (0–10). Secondary outcome measures included: operative time (Recorded from skin incision to closure) and complications, particularly the occurrence of Popeye deformity (clinical observation of a bulge in the anterior arm during elbow flexion) and other surgery-related adverse events (e.g., infection, nerve injury, anchor failure).

### 2.4 Statistical analysis

Statistical analysis was performed using SPSS software (version 23.0). Continuous variables (e.g., scores, operation time) were presented as mean ± standard deviation and compared using independent samples *t*-test or Mann–Whitney *U*-test between groups, and repeated-measures analysis of variance (*ANOVA*) or Friedman test within groups across different time points. Categorical data (e.g., occurrence of Popeye deformity, gender) were presented as frequencies and percentages and compared using the chi-square test or Fisher's exact test. A *p*-value of less than 0.05 was considered statistically significant.

## 3 Results

### 3.1 Patient demographics

A total of 63 patients (39 men, 24 women) with a mean age of 67.3 years (range: 60–76 years) were included. Twenty-nine tears involved the left shoulder and 34 the right shoulder. No significant differences were observed between the isolated tenotomy group (*n* = 28) and the tenotomy with tenodesis group (*n* = 35) regarding baseline characteristics, including age, gender distribution, affected shoulder, and preoperative functional scores (ASES, Constant-Murley) and VAS pain scores (all *p* > 0.05), indicating comparability between the groups (Tables 1, 2).

**Table 1:** Comparison of baseline data between the two groups of patients (*n* = 63).

Variable	Control group (*n* = 28)	Study group (*n* = 35)	Test statistic (t or *χ* ^2^)	*P*-value
Age (years,`c ± s)	68.57 ± 6.64	67.25 ± 6.20	0.810	0.421
Sex (n, %)			0.263	0.608
Male	17 (60.7)	19 (54.3)		
Women	11 (39.3)	16 (45.7)		
Disease duration (years)	7.39 ± 2.15	7.26 ± 2.23	0.244	0.808
Affected shoulder			0.013	0.91
Right	14 (50)	17 (48.6)		
Left	14 (50)	18 (51.4)		
Deltoid muscle atrophy	39.5 ± 2.81	40.31 ± 2.55	−1.203	0.234

**Table 2:** Comparison of clinical outcomes between tenotomy and tenodesis groups (mean ± SD).

Group	ASES			VAS			Constant-Murley scores		
	Tenotomy	Tenodesis	*P*	Tenotomy	Tenodesis	*P*	Tenotomy	Tenodesis	*P*
Pre-operation	71.3 ± 8.54	71.1 ± 11.04	0.60	6.6 ± 1.87	6.6 ± 1.6	0.87	66.0 ± 8.5	68.9 ± 9.2	0.21
3 months postoperatively	77.9 ± 8.0	73.0 ± 9.72	0.03*	3.5 ± 1.8	4.3 ± 1.18	0.02*	74.8 ± 8.4	70.4 ± 7.41	0.0322*
6 months postoperatively	79.7 ± 7.79	77.1 ± 9.60	0.261	3.1 ± 1.66	3.85 ± 1.40	0.087	76.5 ± 8.3	73.3 ± 8.92	0.153
12 months postoperatively	80.1 ± 7.34	78.1 ± 8.71	0.33	2.6 ± 1.37	2.5 ± 1.2	0.76	79.0 ± 8.7	78.7 ± 8.93	0.876

ASES, American Shoulder and Elbow Surgeons; VAS, Visual Analog Scale.

* *P* < 0.05.

### 3.2 Functional outcomes and pain assessment

Both surgical techniques led to significant improvements (*p* < 0.05) in ASES scores, Constant-Murley scores, and VAS pain scores at all postoperative follow-up intervals (3, 6, 12 months, and latest follow-up) compared to preoperative values (Table 2).

Early Postoperative Period (3 months): The isolated tenotomy group demonstrated statistically significant better improvement in VAS pain scores (*p* < 0.05) compared to the tenodesis group. Trends towards better ASES and Constant-Murley scores were also observed in the tenotomy group at this early stage, although the differences were not statistically significant (*p* > 0.05) (Table 2).

At the 6-month and 12-month follow-up: The differences in functional scores (ASES, Constant-Murley) and VAS pain scores between the two groups were no longer statistically significant at the 6-month and 12-month evaluations (*p* > 0.05) (Table 2). The above results indicated that both groups maintained their improvements over time.

### 3.3 Operative time and complications

The mean operative time was significantly shorter (*p* < 0.05) for the isolated tenotomy group (87.5 ± 12.6 min; range: 75–104 min) compared to the tenotomy with tenodesis group (99.4 ± 18.1 min; range: 92–153 min).

Regarding complications, Popeye deformity was observed in 4 out of 28 patients (14%) in the isolated tenotomy group during follow-up. No Popeye deformity was observed in the tenodesis group (0/35), and this difference was statistically significant (*p* = 0.0344, *χ* ^2^ = 5.339). No other major complications (e.g., neurovascular injury, deep infection, or anchor failure related to the tenodesis) were reported in either group during the study period.

## 4 Discussion

This study compared the clinical outcomes of two common techniques, isolated tenotomy *vs.* tenotomy with tenodesis, for managing lesions of the LHBT concomitant with RCTs in elderly patients undergoing arthroscopic repair. Our key findings indicate that while both procedures led to significant and comparable improvements in functional scores (ASES, Constant-Murley) and pain (VAS) at 6 and 12 months, differences were observed in the early postoperative period and in specific complication profiles. Specifically, the isolated tenotomy group demonstrated superior pain relief and functional scores at the 3-month mark, likely attributable to earlier initiation of rehabilitation without the constraints of protecting a tenodesis repair. However, this group had a significantly higher incidence of Popeye deformity (14% vs. 0%). Conversely, the tenodesis group required a longer operative time but effectively prevented cosmetic deformities.

RCTs are a predominant source of shoulder pain and dysfunction, particularly in the elderly population (9). The intimate anatomical relationship between the rotator cuff and the LHBT means that pathologies often coexist. There is growing consensus that a diseased LHBT can be a significant source of persistent pain even after otherwise successful rotator cuff repair (10). The mechanisms of LHBT-related pain are multifaceted. As suggested by Takahashi et al. (11), tendon hypertrophy and hypervascularization within the bicipital groove may contribute, while other theories implicate its role in anterior shoulder stability and inflammatory mediators.

Consequently, addressing the LHBT during arthroscopic cuff repair has become a common practice (1). Our findings align with previous research, such as a Chinese meta-analysis, which concluded that both tenotomy and tenodesis effectively alleviate pain and improve function in patients with LHBT lesions accompanied by RCTs, with no significant differences observed in most functional outcome scores (ASES, UCLA, and VAS), shoulder range of motion, or arm strength between the two techniques (12). The central dilemma for surgeons, therefore, lies not in whether to treat the LHBT, but how to best manage it, weighing the advantages and drawbacks of each technique against individual patient factors.

The observed advantage in early recovery (3-month scores) for the tenotomy group is a crucial consideration for elderly patients for whom rapid pain reduction and regaining independence in activities of daily living (ADLs) are paramount. Tenotomy is a simpler, faster procedure, as confirmed by our significantly shorter operative times (13). It avoids potential complications associated with implants, such as anchor pull-out or irritation, and reduces surgical costs. This expediency can be particularly beneficial in reducing anesthesia time for older patients with comorbidities. However, the principal trade-off for these benefits is the risk of Popeye deformity and potential muscle cramping (14), as seen in our study (14%) and others, where rates can be higher. While often cited as a cosmetic concern, it can occasionally be associated with muscle fatigue or cramping. Our tenodesis group successfully avoided this complication, consistent with the literature which shows tenodesis significantly reduces its incidence. It is worth noting that the functional impact of Popeye deformity on elbow flexion strength in the elderly population, whose demands may be less strenuous, is often debated and may be less critical than in younger, more active individuals (15).

The convergence of functional outcomes between the two groups by the 6 and 12-month follow-ups is a highly consistent finding across the literature. This can be explained by the natural history of recovery after arthroscopic cuff repair. Research indicates that shoulder range of motion improves gradually, with significant gains typically occurring after the 3-month mark and continuing to improve up to 12 months postoperatively (16). The initial delay in the tenodesis group's recovery is likely due to the need for a period of protection to allow the fixed tendon to heal to bone, temporarily limiting active motion and strengthening exercises. Once this healing is achieved and rehabilitation intensifies, patients in the tenodesis group catch up functionally. This suggests that the choice of procedure may influence the trajectory of recovery rather than its ultimate endpoint.

When deciding on a surgical strategy, several other factors must be integrated into the clinical decision-making process, including irreparable tears, the role of biological adjuvants, and timing of surgery (17). For massive, irreparable RCTs, alternative and innovative techniques are emerging. The recent development of a functionally graded scaffold that mimics the native tendon-bone interface shows promise in enhancing healing strength and reducing re-tear rates in animal models (18). Furthermore, the use of a biodegradable subacromial spacer balloon has been reported as a viable option to restore biomechanical balance and alleviate pain in such complex cases, offering a new solution for patients who are not candidates for traditional repair or reverse shoulder arthroplasty (19). The use of platelet-rich plasma (PRP) is being increasingly explored to enhance healing after cuff repair (20). Some studies suggest that PRP, particularly leukocyte-poor PRP (LP-PRP), can improve functional scores and reduce postoperative re-tear rates by modulating inflammation and promoting regeneration (21). However, the evidence remains heterogeneous, with significant variations in preparation methods, classification, and application techniques. A critical review also highlights the issue of “spin bias” in systematic reviews on PRP, urging cautious interpretation of positive findings (22). Future standardized research may clarify its role as an adjunct to both tenotomy and tenodesis. For traumatic massive RCTs, evidence suggests that delaying surgery beyond six months may increase the risk of re-tear. Although this directly concerns cuff repair integrity, it underscores the importance of a comprehensive and timely surgical approach, which includes a plan for the LHBT.

### 4.1 Limitations and future directions

Our study has several limitations common to retrospective analyses, including potential selection bias. As a retrospective analysis, the sample size was inherently determined by the number of eligible patients who underwent surgery at our institution during the defined study period (January 2020 to December 2022) and met all inclusion and exclusion criteria. Our primary aim was to conduct an initial comparative evaluation of these two surgical techniques within our specific patient population. However, since the sample size cannot be altered in this retrospective study, the potential for Type II errors should be acknowledged due to the insufficient sample size. The follow-up period of 12 months, while adequate to capture primary functional outcomes, is insufficient to assess long-term consequences such as the persistence of Popeye deformity, the durability of tenodesis fixation, or the progression of any cuff re-tears. Larger, prospective, randomized controlled trials with longer follow-up are needed to validate these findings.

Future research should also focus on refining patient selection criteria. For instance, younger, more active patients, or those in whom cosmesis is a high priority, might benefit more from tenodesis despite the longer initial recovery. In contrast, for older, lower-demand patients, the faster recovery and simplicity of tenotomy might make it the preferred choice, accepting a small risk of cosmetic deformity. Techniques like the “Chinese way”, which offers different procedural types based on intraoperative findings of LHBT quality, also represent a move towards more personalized surgical strategies.

## 5 Conclusion

In conclusion, both isolated tenotomy and tenotomy with tenodesis are effective and viable surgical strategies for managing LHBT lesions in conjunction with arthroscopic rotator cuff repair in elderly patients. Isolated tenotomy offers the advantages of a shorter operation and significantly faster early pain relief and functional recovery, making it an excellent option for low-demand patients prioritizing a quick return to function. Tenodesis, while requiring a longer operative time and a potentially slower initial recovery, effectively prevents Popeye deformity and provides equivalent functional outcomes by 6 to 12 months. The choice between these procedures should not be based on a perceived superiority of one over the other but should be individualized, considering the patient's age, activity level, cosmetic concerns, and the surgeon's expertise, within a shared decision-making process.

## Data Availability

The raw data supporting the conclusions of this article will be made available by the authors, without undue reservation.

## References

[1] Mzeihem M Nassereddine M El ZA Amirouche F Elhassan B. Exploring superior capsular reconstruction and tendon transfers for massive irreparable posterosuperior rotator cuff tears. EFORT Open Rev. (2025) 10(9):660–70. 10.1530/EOR-2024-0139 40905927 PMC12412289

[2] Miranda LA Kim BT Llinas PJ Baek CH Werthel JD Kany J. Combined Latissimus dorsi/Teres Major transfer and superior capsular reconstruction using autogenous Biceps tendon effectively relieves pain and improves shoulder function for posterosuperior irreparable rotator cuff tears. Arthrosc Sports Med Rehabil. (2025) 7(3):101147. 10.1016/j.asmr.2025.101147 40692922 PMC12276545

[3] Ho NS Le NNT Nguyen PD Tang A Tran DH. Superior capsular reconstruction using the long head of the Biceps tendon for large to massive rotator cuff tears with pseudoparalysis: a prospective clinical study. Int Orthop (2025) 49(9):2123–30. 10.1007/s00264-025-06612-2 40664842

[4] Vigie R Bonnevialle N Hao KA Berhouet J Charousset C. Tenotomy or tenodesis versus conservation of the long head of the biceps tendon in the repair of isolated supraspinatus tears: a systematic review of the literature. Orthop Traumatol Surg Res. (2023) 109(8S):103673. 10.1016/j.otsr.2023.103673 37657502

[5] Luo JL Huang YB Deng XH Wang JS Li YH Li WP The long head of Biceps tendon tenotomy in idiopathic glenohumeral adhesive capsulitis surgery improves early rehabilitation outcomes. Orthop Surg. (2025) 17(8):2331–41. 10.1111/os.70104 40579784 PMC12318667

[6] Forsythe B Gamsarian V Patel HH Berlinberg E Warrier A Goheer H Arthroscopic Biceps tenodesis with interference screw fixation: a technique video. Video J Sports Med. (2024) 4(4):26350254241230972. 10.1177/26350254241230972 40309052 PMC11752396

[7] Henssler L Zeman F Akgun D Thiele K Pauly S Greiner S Implant-free loop tenodesis compared to arthroscopic anchor tenodesis for the treatment of long head of biceps tendon disorders (LOOPTEN trial): study protocol for a multi-center non-inferiority randomized controlled trial. BMC Musculoskelet Disord. (2025) 26(1):567. 10.1186/s12891-025-08818-2 40474104 PMC12142869

[8] Worden JA Kopriva JM Gass HM Hussain ZB Karzon AL Chopra KN Surgical treatment of long head of biceps pathology: analyzing trends in the United States from 2010 to 2019. JSES Rev Rep Tech. (2025) 5(2):160–9. 10.1016/j.xrrt.2024.12.013 40321877 PMC12047552

[9] Sabelle A Salle B Charousset C Jacquot A Gadea F Guery J Two-year outcomes of non-conservative treatment of the long head of the biceps tendon in the repair of small supraspinatus tears: a multicenter prospective study. Orthop Traumatol Surg Res. (2025) 104451. 10.1016/j.otsr.2025.104451 41067626

[10] Tsai YC Lin PH Ho CH Chen YT Wang JH. Retrospective study on biceps tendon rerouting and local grafting with coracohumeral ligament for massive rotator cuff repair in Taiwanese population. J. Formos. Med. Assoc. (2025):S0929-6646(25)00520-0. 10.1016/j.jfma.2025.09.036 41033911

[11] Takahashi N Sugaya H Matsumoto M Miyauchi H Matsuki K Tokai M Progression of degenerative changes of the biceps tendon after successful rotator cuff repair. J Shoulder Elbow Surg. (2017) 26(3):424–9. 10.1016/j.jse.2016.09.052 27914841

[12] Shang X Chen J Chen S. A meta-analysis comparing tenotomy and tenodesis for treating rotator cuff tears combined with long head of the biceps tendon lesions. PLoS One. (2017) 12(10):e0185788. 10.1371/journal.pone.0185788 29016616 PMC5633150

[13] Vajda M Szako L Hegyi P Eross B Gorbe A Molnar Z Tenodesis yields better functional results than tenotomy in long head of the biceps tendon operations-a systematic review and meta-analysis. Int Orthop (2022) 46(5):1037–51. 10.1007/s00264-022-05338-9 35254476 PMC9001564

[14] Panico L Roy T Namdari S. Long head of the Biceps tendon ruptures: biomechanics, clinical ramifications, and management. JBJS Rev. (2021) 9(10). 10.2106/JBJS.RVW.21.00092 34695033

[15] Lim TK Moon ES Koh KH Yoo JC. Patient-related factors and complications after arthroscopic tenotomy of the long head of the biceps tendon. Am J Sports Med. (2011) 39(4):783–9. 10.1177/0363546510388158 21212312

[16] Zhang C Yang G Li T Pang L Li Y Yao L Biceps tenodesis better improves the shoulder function compared with tenotomy for long head of the Biceps tendon lesions: a meta-analysis of randomised controlled trials. J Clin Med. (2023) 12(5):175. 10.3390/jcm12051754 PMC10003204 36902540

[17] Garg AK Meena A Farinelli L D'Ambrosi R Tapasvi S Braun S. Partial subscapularis tear: state-of-the-art. J ISAKOS. (2024) 9(6):100290. 10.1016/j.jisako.2024.06.009 38909905

[18] Shi J Yao H Chong H Hu X Yang J Dai X Tissue-engineered collagen matrix loaded with rat adipose-derived stem cells/human amniotic mesenchymal stem cells for rotator cuff tendon-bone repair. Int J Biol Macromol (2024) 282(Pt 4):137144. 10.1016/j.ijbiomac.2024.137144 39488324

[19] Lobao MH Canham RB Melvani RT Abboud JA Parks BG Murthi AM. Biomechanics of biodegradable subacromial balloon spacer for irreparable superior rotator cuff tears: study of a cadaveric model. J. Bone Joint Surg. Am. (2019) 101(11):e49. 10.2106/JBJS.18.00850 31169580

[20] Yao L Pang L Zhang C Yang S Wang J Li Y Platelet-Rich plasma for arthroscopic rotator cuff repair: a 3-arm randomized controlled trial. Am J Sports Med. (2024) 52(14):3495–504. 10.1177/03635465241283964 39425250

[21] Peng Y Du L Yang B Fan D Jia S Zheng C. Efficacy of platelet-rich plasma and platelet-rich fibrin in arthroscopic rotator cuff repair: a systematic review and meta-analysis. PM R. (2023) 15(12):1643–53. 10.1002/pmrj.13049 37526570

[22] Moulton SG Hartwell MJ Feeley BT. Evaluation of spin bias in systematic reviews and meta-analyses of rotator cuff repair with platelet-rich plasma. Am J Sports Med. (2024) 52(13):3412–8. 10.1177/03635465231213039 38323324

